# Covering Hierarchical Dirichlet Mixture Models on binary data to enhance genomic stratifications in onco-hematology

**DOI:** 10.1371/journal.pcbi.1011299

**Published:** 2024-02-02

**Authors:** Daniele Dall’Olio, Eric Sträng, Amin T. Turki, Jesse M. Tettero, Martje Barbus, Renate Schulze-Rath, Javier Martinez Elicegui, Tommaso Matteuzzi, Alessandra Merlotti, Luciana Carota, Claudia Sala, Matteo G. Della Porta, Enrico Giampieri, Jesús María Hernández-Rivas, Lars Bullinger, Gastone Castellani, with the HARMONY Healthcare Alliance Consortium

**Affiliations:** 1 IRCCS Istituto delle Scienze Neurologiche di Bologna, Bologna, Italia; 2 Department of Hematology, Oncology and Cancer Immunology, Campus Virchow, Charité – Universitätsmedizin Berlin, corporate member of Freie Universität Berlin and Humboldt-Universität zu Berlin, Berlin, Germany; 3 Department of Hematology and Stem Cell Transplantation, University Hospital Essen, Essen, Germany; 4 Department of Hematology and Oncology, Marienhospital University Hospital, Ruhr-University Bochum, Bochum, Germany; 5 Department of Hematology, Amsterdam UMC location Vrije Universiteit, Amsterdam, the Netherlands; 6 AbbVie Deutschland GmbH & Co KG, Wiesbaden, Germany; 7 Bayer AG, Berlin, Germany; 8 Molecular Genetics in Oncohematology, Institute of Biomedical Research of Salamanca, Salamanca, Spain; 9 Department of Physics and Astronomy, University of Florence, Sesto Fiorentino, Italy; 10 Physics and Astronomy Department, University of Bologna, Bologna, Italy; 11 Department of Medical and Surgical Sciences—DIMEC, University of Bologna, Bologna, Italy; 12 Comprehensive Cancer Center, IRCCS Humanitas Clinical and Research Center and Department of Biomedical Sciences, Humanitas University, Milan, Italy; 13 Hematology Department, University Hospital of Salamanca, Salamanca, Spain; 14 Cancer Research Center of Salamanca, Salamanca, Spain; Universita degli Studi di Torino, ITALY

## Abstract

Onco-hematological studies are increasingly adopting statistical mixture models to support the advancement of the genomically-driven classification systems for blood cancer. Targeting enhanced patients stratification based on the sole role of molecular biology attracted much interest and contributes to bring personalized medicine closer to reality. In onco-hematology, Hierarchical Dirichlet Mixture Models (HDMM) have become one of the preferred method to cluster the genomics data, that include the presence or absence of gene mutations and cytogenetics anomalies, into components. This work unfolds the standard workflow used in onco-hematology to improve patient stratification and proposes alternative approaches to characterize the components and to assign patient to them, as they are crucial tasks usually supported by a priori clinical knowledge. We propose (a) to compute the parameters of the multinomial components of the HDMM or (b) to estimate the parameters of the HDMM components as if they were Multivariate Fisher’s Non-Central Hypergeometric (MFNCH) distributions. Then, our approach to perform patients assignments to the HDMM components is designed to essentially determine for each patient its most likely component. We show on simulated data that the patients assignment using the MFNCH-based approach can be superior, if not comparable, to using the multinomial-based approach. Lastly, we illustrate on real Acute Myeloid Leukemia data how the utilization of MFNCH-based approach emerges as a good trade-off between the rigorous multinomial-based characterization of the HDMM components and the common refinement of them based on a priori clinical knowledge.

## Author summary

Explainable models are particularly attractive nowadays since they have the advantage to convince clinicians and patients. In this work we show that the standard workflow used in onco-hematology that leverages on the Hierarchical Dirichlet Mixture Model (HDMM) can benefit from the usage of alternative statistical approaches to better model genomics data. First, HDMMs are typically utilized to cluster the presence or absence of genomic alterations into components. Second, the components are characterized by genomic drivers according to the HDMM outcome, along with to clinical considerations, and eventually patients stratification is performed based on the sole occurrence of the drivers. Here, we present a multinomial-based approach and a Multivariate Fisher’s Non-Central Hypergeometric (MFNCH) based approach to tackle components characterization and patients stratification. This work highlights that the MFNCH-based approach performs as good as, if not better, than the multinomial-based approach on simulated data. At the same time the characterization provided by the MFNCH-based approach on real data succeeds to outline all genomic drivers suggested by the standard workflow, which is a result that the rigorous multinomial-based approach fails to accomplish.

This is a *PLOS Computational Biology* Methods paper.

## Introduction

In medicine, onco-hematology is leading the way towards personalized medicine thanks to the efforts dedicated to characterize and to cluster the genomic nature of blood tumors. Clustering is a paramount task carried out in several fields of data-driven science [1]. The ability to organize data in meaningful groups opens the possibility to identify shared characteristics that bring observations together and discriminate others that push them apart. Better knowledge of such characteristics contributes to better profile observations and to draw observation-specific considerations, i.e., personalization. To perform clustering, nonparametric Bayesian methods are becoming of paramount importance in several field of medicine, including cancer research [2]. Especially with categorical data, Hierarchical Dirichlet Mixture Models (HDMMs), which are notorious nonparametric Bayesian methods, are quickly gauging interest [3–7]. Their usage already proved to boost the definition of new clinical classification systems and the discovery of unknown groups of co-occurrent genomic alterations. The HDMMs have also been used transversally on genomic data, including copy number variation plus transcriptomic integration [8], pan-cancer proteomic characterization [9], cancer subtyping with microRNA [10] and disease classes discrimination based on genomics, transcriptomics and epigenomics [11].

In this paper we address the standard workflow in onco-hematology that makes use of the HDMM to investigate the genomics panorama of blood cancers. In onco-hematology, a well-defined clinical problem is to stratify patients based on their altered genomics pattern to enhance diagnostic precision and prognostic power. The HDMM is used to cluster genomic information, usually represented as the presence or absence of genomic alterations, i.e., gene mutations and cytogenetics anomalies. The objects of the clustering are then binary variables and to understand how they can be clustered using HDMMs some key considerations are made. Frequently, in fact, binary data are viewed as count data and, as such, they are modelled by multinomials. Although a vector of binary values can be sampled from multinomials, sampling from multinomials does not guarantee to yield a vector of binary values. This is because the multinomial distribution models samplings with replacements, i.e., an observation can be drawn many times, and its parameters are probabilities, which exactly expresses that some observations are more probable than others. To address this caveat, the simplest solution is to assume that a binary vector can derive from a sampling without replacement. This case is modelled by the well-known multivariate hypergeometric distribution. Since the parameters of the multivariate hypergeometric distribution are the number of times an observation can be sampled, if all parameters are set to one, the domain of the distribution is only composed by binary data. That is, the multivariate hypergeometric distribution can be tailored to produce only binary data. Though, differently from multinomials, the multivariate hypergeometric distribution, with all its parameters set to one, does not provide ways to prioritize observations, which means that only one hypergeometric distribution would exist. Since this would prevent the usage of a mixture model, an intuitive solution would be to exploit a distribution modelling sampling without replacement but with prioritized observations. This case falls under the domain of the so-called Multivariate Non-Central Hypergeometric (MNCH) distributions, that endow each observation of the multivariate hypergeometric distribution with an additional parameter (see S1 Data). The larger the weight of an observation, the higher it is the bias of its occurrence. In this work the usage of the MNCH distribution formulated by Fisher (MFNCH) [12, 13] on the components found by the HDMM reports promising results in support of patients stratification.

**Fig 1:**
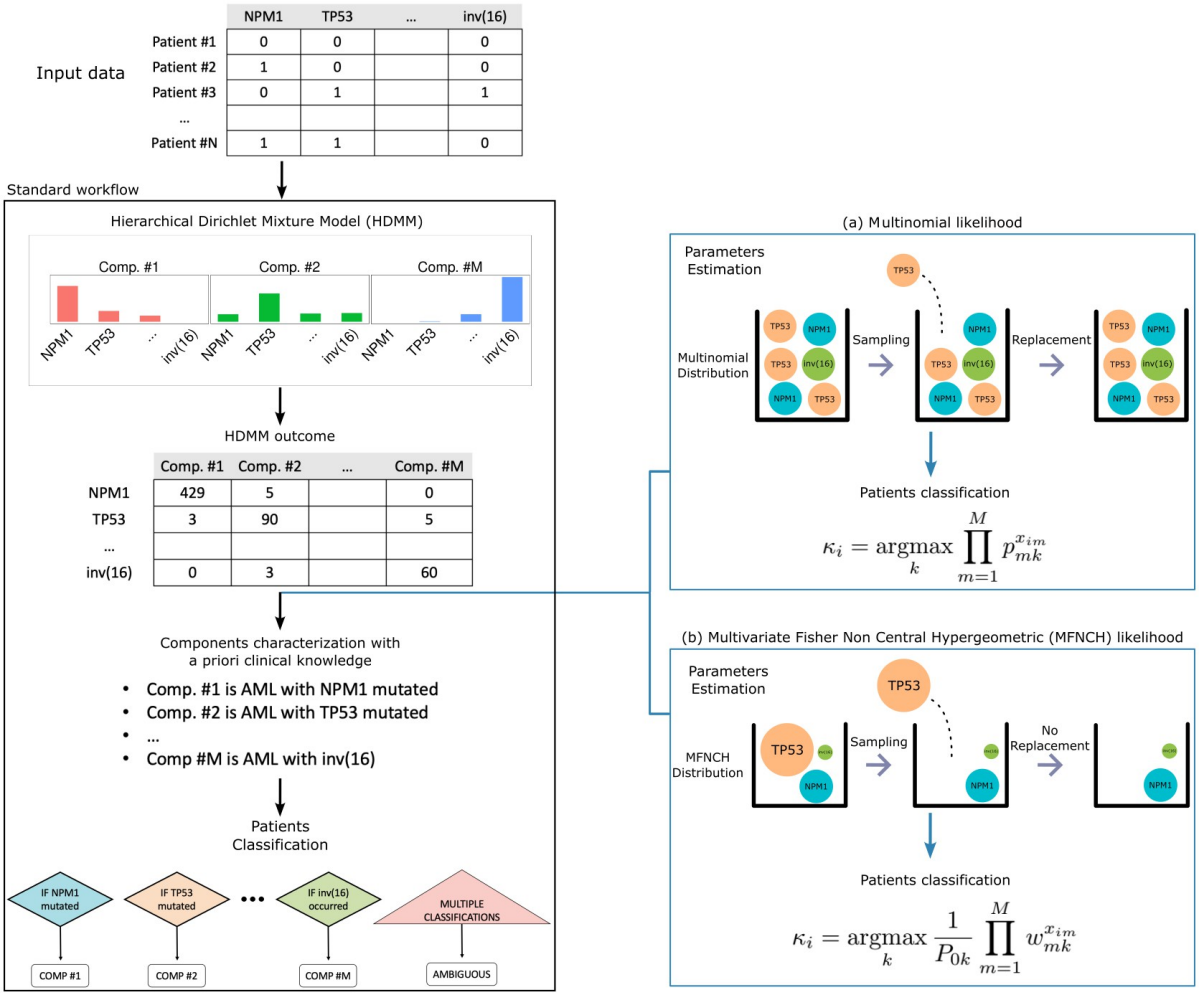
Illustration of the herein proposed approaches to enhance the standard workflow for onco-hematological patients stratification. Description of the common recent workflow in onco-hematology (in the black box) for the analysis of genomic data used to improve the identification of disease components that can potentially support the progress of novel disease classification systems. On the right, we illustrate the herein presented novel approaches to enhance the statistical characterization of components and to provide a maximum-likelihood inspired alternative to perform patients classification. Our approaches are built upon the outcome of the Hierarchical Dirichlet Mixture Model (HDMM) of multinomials that usually fits the data. Given the HDMM outcome, the components are usually characterized by a single or a few genomic drivers inspired by how the HDMM clustered the genomic alterations and by a priori clinical knowledge. In contrast, our approaches utilize the HDMM outcome to respectively characterize the components either as multinomials, i.e., in line with the HDMM, or as Multivariate Fisher Non Central Hypergeometric (MFNCH) distributions. Each distribution models a different urn problem. The multinomials model drawings with replacement from an urn with multiple marbles with different colors. Instead, the MFNCH distributions we use model drawings without replacement from an urn with one single marble per color and with each marble with a different size.

Our work builds upon the standard workflow of HDMMs in onco-hematology and proposes to change part of it (Fig 1). The input data, i.e., a matrix of patients and genomic alterations respectively along rows and columns, is modelled by a HDMM of multinomials and all patients alterations are clustered into components. The HDMM determines the number of components autonomously, which is the main appealing reason of its recent popularity. The outcome of the HDMM is a matrix of genomic alterations and components (along rows and columns) that express how alterations were clustered across components. At this point, each component gets usually characterized by genomic drivers, which represent frequent genomic alterations in a component that also carry clinical value. The ultimate idea is that a patient is assigned to a component only if it harbors its driver (or drivers). In cases of multiple assignments, a patient is usually considered ambiguous. Therefore, from the characterization of components to patients assignments, the statistical support provided by the HDMM is used partially. Here, we propose to change these two steps of the workflow as follows: (i) we consider each component as a statistical distribution and we estimate its parameters; (ii) we assign a patient to its most likely component (i.e., the component with the largest likelihood of observing that patient). As shown in Fig 1, we consider components either as multinomials, which is exactly what the HDMM implicitly fits, or as MFNCH distributions. In this work, we simulated data to assess and compare the multinomial-based approach with the MFNCH-based approach, where we found the latter to be the best compromise for stratification use. Plus, since the HDMM fit crucially influences the whole workflow final result we provide additional insights on the convergence of HDMM, which we found to be a hardly met condition. On top of that, we showed the results of our approaches in a real case scenario of Acute Myeloid Leukemia (AML), where the standard workflow was applied and was crucially used to support the new classification of AML [3]. Even on this real data, the MFNCH-approach showed to be an insightful compromise between the thorough statistical multinomial-approach and the clinically-oriented standard workflow.

## Materials and methods

### Mixture models

Mixture models enable to model observations as a combination of multiple distributions [14]. An observation *x*_*n*_ for a sample *X*_*n*_ is usually supposed to be drawn from a single probability density function *f*(*X*_*n*_ = *x*_*n*_). Mixture models, instead, assume an observation to be sampled from *G* components (or groups) with prior probabilities {*π*_*g*_}_*g*=1‥*G*_ and with conditional densities *f*_*g*_(*X*_*n*_ = *x*_*n*_|*π*_*g*_). The density function for sample *X*_*n*_ is the marginal density:

$$\begin{array}{c} f\left(X_{n}=x_{n}\right)=\sum_{g}^{G}f_{g}\left(X_{n}=x_{n}|Z_{n}=g\right)\pi \left(Z_{n}=g\right)\;\;, \end{array}$$ (1)

where the proxy variable *Z*_*n*_ indicates the component an observation is drawn from. Upon drawing from the mixture model, the proxy *Z*_*n*_ becomes a binary random variable that is zero for all component except being one for a single *g*-th. Therefore *Z*_*n*_ can be replaced by a random vector ${\vec{Z}}_{n}$ whose observations are single trials from a multinomial distribution with parameters equal to the prior component probabilities. That is,

$$\begin{array}{c} \left(Z_{n1}=z_{n1},‥,Z_{nG}=z_{nG}\right)∼\text{Multi}\left(\pi_{1},‥,\pi_{g}\right)\;\;. \end{array}$$ (2)

Thus, the sampling procedure from a mixture model first requires to draw a *g*-th component according to (2) and, secondly, it requires to sample from the corresponding density *f*_*g*_(⋅). In onco-hematology the widespread assumption is that patients’ genomic data derives from a mixture of multinomial components, so that the parameters of the density functions are probabilities.

### Dirichlet Mixture Model

Dirichlet Processes (DPs) [15] can be seen as the a priori processes of mixture models. The study of DPs usually involves other processes such as the Chinese Restaurant Process (CRP) [16] and the Blackwell-MacQueen (BM) models [17]. These processes fall outside the scope of this work but ultimately DP generalizes them both. Now, if a probability distribution *H* over the parameters of the components is defined, it is immediate to determine which parameters are more common, or which component is. Though, *H* is one among many potential distributions and its choice limit the exploration of the parameters space. What the DPs address is to provide a statistical framework to handle many possible a priori distributions. In other words, a DP is a probability distribution over the possible probability distributions for the parameters, which we generally refer to as *ϕ*_*k*_. Formally,

$$\begin{array}{c} G∼\text{DP}(\gamma ,H) \end{array}$$ (3)

means that

$$\begin{array}{c} \left(G\left(\phi_{1}\right),‥,G\left(\phi_{K}\right)\right)∼\text{Dir}\left(\gamma H\left(\phi_{1}\right),‥,\gamma H\left(\phi_{K}\right)\right)\;\;. \end{array}$$ (4)

From a discrete perspective the above formulation implies that,

$$\begin{array}{c} G=\sum_{k=1}^{K}w_{k}\delta_{\phi_{k}}\left(A\right)\;\;, \end{array}$$ (5)

where the weights represent probabilities, i.e., *w*_*k*_ = *G*(*ϕ*_*k*_), and as such $\sum_{k=1}^{K}w_{k}=1$. Since there is no restriction on *K* the number of components can be infinite (*K* = ∞). Though, the number of components is never infinite, which implies that only a few components in the mixture have a non-zero probability of occurrence. This fact is usually described by processes with rich components that progressively get richer. All these concepts boil down to the complete formulation of the Dirichlet Mixture Model (DMM), which is described by:

$$\begin{array}{c} G∼\text{DP}(\gamma ,H) \end{array}$$ (6)

$$\begin{array}{c} {\vec{\phi }}_{n}|G\overset{iid}{∼}G\;\;\text{for}\;\;n=\left[1,N\right] \end{array}$$ (7)

$$\begin{array}{c} x_{n}|{\vec{\phi }}_{n}\overset{ind}{∼}f\left({\vec{\phi }}_{n}\right)\;\;\text{for}\;\;n=\left[1,N\right]\;\;, \end{array}$$ (8)

where iid stands for identically and independently distributed. In the onco-hematological scenario the HDMM intends to potentially cluster patients in infinite components, whose parameters are typical probabilities for the multinomials. During the Dirichlet Mixture Model (DMM) fit, the underlying “rich get richer” mechanism consents to automatically detect a probability distribution *G* of parameters. Since the majority of possible parameters have zero probability, i.e., *w*_*k*_ = 0, using a DP a priori gives the benefit of not choosing the number of expected components.

### Hierarchical Dirichlet Mixture Model

The DMM can be further extended by other DPs when observations are believed to be organized in groups [18]. Following the previous formulation, a realization from a Hierarchical Dirichlet Mixture Model (HDMM) follows:

$$\begin{array}{c} G_{0}∼\text{DP}\left(\gamma ,H\right) \end{array}$$ (9)

$$\begin{array}{c} G_{m}∼\text{DP}\left(\alpha ,G_{0}\right) \end{array}$$ (10)

$$\begin{array}{c} {\vec{\phi }}_{n,m}|G_{m}\overset{iid}{∼}G_{m}\;\;\text{for}\;\;n=\left[1,N_{m}\right] \end{array}$$ (11)

$$\begin{array}{c} x_{n,m}|{\vec{\phi }}_{n,m}\overset{ind}{∼}f\left({\vec{\phi }}_{n,m}\right)\;\;\text{for}\;\;n=\left[1,N_{m}\right]\;\;. \end{array}$$ (12)

Typically, the modelling of onco-hematological genomic data is performed with a HDMM. In this case, the objects clustered by the HDMM are the single genomic alterations of each patient and not the whole patients with their co-existing sets of genetic alterations. With this perspective change each patient is considered as a separate group of genomic alterations. The main difference w.r.t. the DMM is that each patient is endowed with its own a priori probability distribution of parameters and an alteration is clustered also based on the patient it belongs to. Clearly, multiple levels can be added to the HDMM structure and the greater the number of levels (e.g. gender, age, etc.), the greater will be the sensitivity of the fit to retrieve group-related patterns.

### Extracting components

Once the statistical foundation of the HDMM is set and datasets with no-missing value are prepared, the HDMM needs to be fitted. Usually, Gibbs sampling [19] is employed and assigns, at each step of such Markov Chain Monte Carlo (MCMC) the observations, e.g. every genomic alteration in each patient, to a component one by one. Along the MCMC the number of components changes based on the observations they were assigned to in each previous step. Usually the MCMC is set to start from completely random components and the first iterations are excluded to first reach convergence. Upon convergence, every iteration along the MCMC estimates how observations are clustered and how many components exist. Hence, we obtained a sample from a HDMM as a matrix counting the number of times an observation is assigned to each component. Though, since many iterations compose a MCMC, we collected a matrix of this kind at every step. Additionally, multiple MCMCs can be optionally run to address the dependency of the HDMM samples w.r.t. to the starting random sampling, which expands the collection of matrix samples. Eventually, we used all these matrix samples to determine the average number of times an observation belongs to every component. This approach is the standard one in onco-hematological studies to determine groups (i.e., components) of genomic alterations and is based on the assumption that the data can be represented as a mixture of multinomial distributions, which shows how frequent each genomic alteration is clustered in every component. This popular assumption is supported by the ability of using the multinomial parameters to prioritize genomic alterations within components (i.e., ranking alterations based on their frequencies) and the feasibility of fitting a HDMM of multinomials on count data, which for other distributions may require the computation of partition functions across samplings. To better adapt to the binary nature of the input data and to increase the importance of discriminative genomic alterations between components, we alternatively utilized the Fisher’s Non-Central Hypergeometric distribution (MFNCH) (see more details in S1 Data). We remark that we estimated, from the components obtained by the HDMM, the parameters of MFNCH distributions. In other words, we questioned whether representing the mixture of multinomials found by the HDMM as mixture of MFNCH distributions could provide an insightful characterization of the components in order efficiently assign patients. To our knowledge this is the first time the MFNCH distribution is used to better characterize clusters derived from the HDMM. Therefore, we eventually modelled the data as a mixture of MFNCH distributions. To estimate the MFNCH weights for each observation we adopted the Cornfield’s approximation [20] after estimating the observation mean (e.g. average occurrences for a genomic alteration in a component). Further details of components extraction can be found in the S1 Data.

### Data simulation

We attempt to simulate onco-hematological data using MFNCH components to generate simulated patients. To determine the weights of the MFNCH components, we sampled them from a Dirichlet distribution. Several mixtures of MFNCH components were then generated to cover several possible scenarios in onco-hematology. We used four variables to control the simulations. The first variable, *K*, was the number of components underlying the mixture and two cases were taken into account, i.e., *K* = 5, 10. The number of simulated components were chosen consistently with the number of components found by previous studies on real data (i.e., # components ≤ 10). The second variable *α*_*sim*_ regulated the concentration parameters of the a priori Dirichlet distribution that determine the weights of the components. Two extreme situations were studied: *α*_*sim*_ = 1 and *α*_*sim*_ = 1/*M*, where *M* stands for the number of simulated genomic alterations. The former yields MFNCH components with almost uniform weights, which entails that the MFNCH components of the mixture tend to be similarly prioritize alterations. The latter, contrarily, rendered MFNCH distributions with a few high weighted and many low weighted alterations, which increased the heterogeneity of prioritizations across components. The third variable was the average number of alterations per simulated patient, which was ranged from one to ten. This short low range was chosen to be in the order of magnitude of the number of average alterations per patient found in real onco-hematological datasets, as that is hardly greater than five with the usual gene panels. Following data simulation, we ran the usual first part of the standard workflow by fitting a HDMM of multinomials. Therefore we did not change the HDMM common usage. Multinomials eventually remained the underlying distribution of the mixture to be consistent with the current state-of-the-art in onco-hematological studies and because the alternative MFNCH distribution is not a feasible solution. In fact, changing the HDMM to directly model a mixture MFNCH turns the fit into a time-consuming task due to the computation of the partition functions of the distributions that makes their implementation slow and hardly scalable. Along with these three variable, the HDMMs were also provided with a concentration parameter *α*_*HDMM*_. The influence of this variable was additionally observed as for *α*_*sim*_, i.e. either *α*_*HDMM*_ = 1 or *α*_*HDMM*_ = 1/*M*. In fact, when *α*_*HDMM*_ = 1 the HDMM favors the process to potentially find uniform-like components. In contrast, when *α*_*HDMM*_ = 1/*M* the HDMM searches for low-overlapping components. The combinations of all four variables allowed to dissert the modelling of the simulated patients from different standpoints. In total, we ended up with forty simulated datasets, each including one thousand simulated patients (*N* = 1000) featuring fifty (*M* = 50) simulated genomic alterations. Half of the datasets were drawn from five MFNCH distributions (*K* = 5), while the others from ten (*K* = 10). Plus, these MFNCH distributions were generated according to either a concentration parameter *α*_*sim*_ equal to one or to 1/*M*. Datasets further differed based on the average number of simulated genomic alterations per patient that spanned from one to ten. Each of the forty datasets was modelled by two HDMMs: one driven by a concentration parameter *α*_*HDMM*_ of one or 1/M. Therefore eigthy HDMMs were run and ten MCMCs were run for each of them to gather ten independent estimates, resulting in 800 running MCMCs.

### Patients stratification

The first part of the standard workflow uses HDMM to cluster genomic alterations. The second part then usually characterize each component based on some frequent and clinically relevant genomic drivers to eventually assign patients according to the drivers they carry. Statistically, though, each component of the HDMM is a multinomial whose parameters can be estimated based on how the genomic alterations clustered. Therefore, we propose to estimate the parameters of the multinomials and use them to assign a patient to the component maximizing the likelihood of generating that patient. We utilize the probability mass function of the multinomial to calculate the likelihoods. In statistical terms, once the *K* components mixture and their multinomial parameters ${\vec{p}}_{k}$ are estimated, we assign a patient to the component with the highest p.m.f. value:

$$\begin{array}{c} \kappa_{i}=\underset{k}{\text{argmax}}\prod_{m=1}^{M}p_{mk}^{x_{im}}\;\; \end{array}$$ (13)

where *κ*_*i*_ is the component that the *i*-th patient is assigned to. The *x*_*ij*_ represents the presence or absence of the *j*-th genomic alteration for the *i*-th patient. To perform patient stratification Eq 13 does not use the partition function of the multinomials since it is the same for all. We alternative propose to use the clustering of the genomic alterations provided by the HDMM to characterize components as MFNCH distributions and to estimate their parameters ${\vec{w}}_{k}$ accordingly (see Supplementary Material for further details). It follows that to stratify patient across components the same maximum-likelihood approach applies. That is:

$$\begin{array}{c} \kappa_{i}=\underset{k}{\text{argmax}}\frac{1}{P_{0k}}\prod_{m=1}^{M}w_{mk}^{x_{im}}\;\;, \end{array}$$ (14)

where *P*_0*k*_ is the partition function for the *k*-th component.

## Results

### HDMM convergence on simulated patients

**Fig 2:**
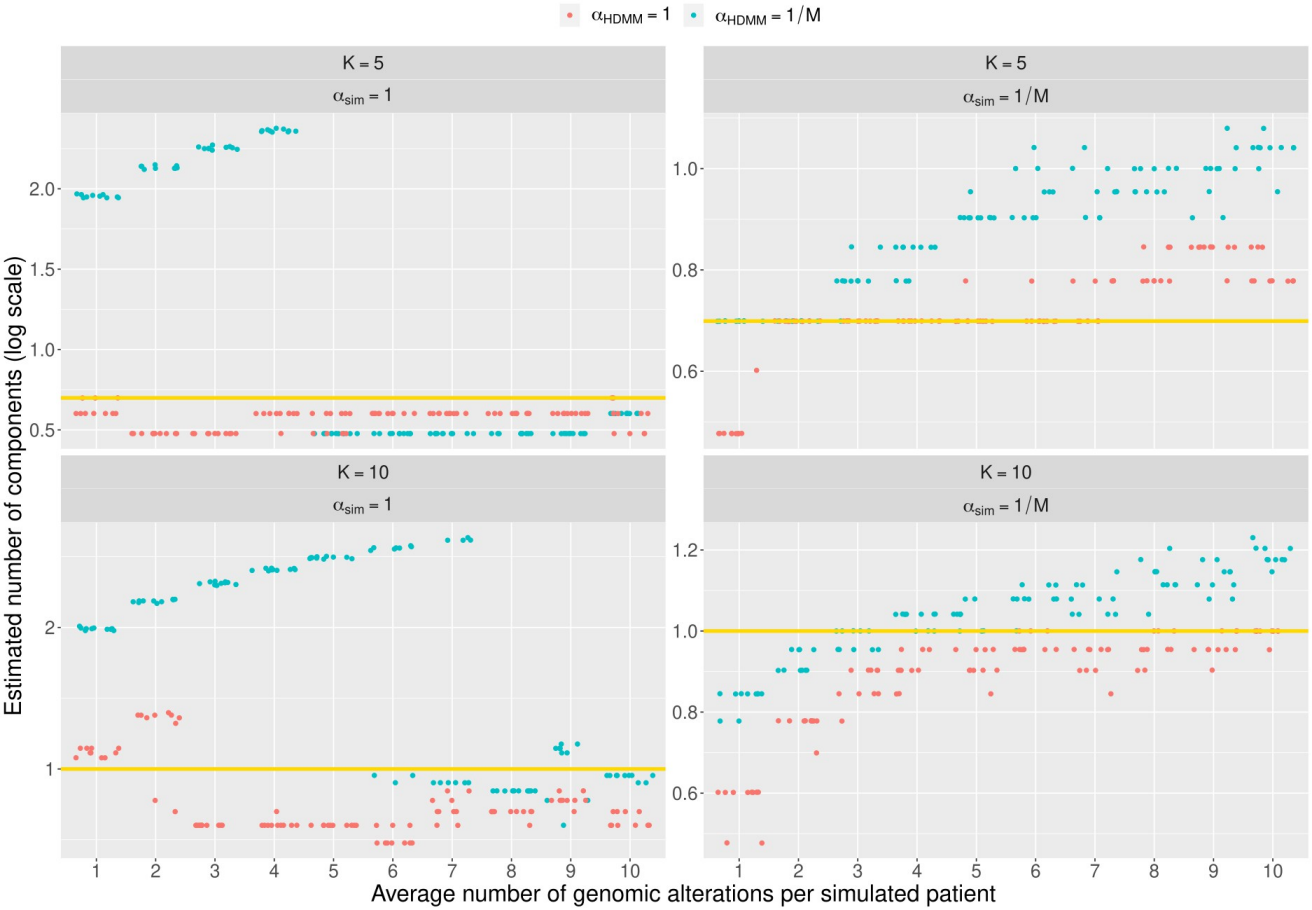
Difficulty for HDMM to estimate the expected number of components on simulated patients. Results on the capability of convergence of the HDMM on simulated data that aim to reproduce the onco-hematological data commonly used to discover novel disease classes. In this plot we observe the number of components estimated by the HDMM (y-axis) along the average number of genomic alterations per simulated patient for several settings (x-axis). The expected *K* number of components in logarithmic scale is indicated by the horizontal yellow line, while the observed number is reported on the y-axis. Along with *K*, each quadrant shows whether the simulated components tend to be uniform-like (*α*_*sim*_ = 1) or low-overlapping (*α*_*sim*_ = 1/*M*, where *M* is the number of genomic alterations). Plus, the color of the points represent if the HDMM was run to detect more uniform-like components (*α*_*HDMM*_ = 1, in red) or more disjunct components (*α*_*HDMM*_ = 1/*M*, in blue).

**Fig 3:**
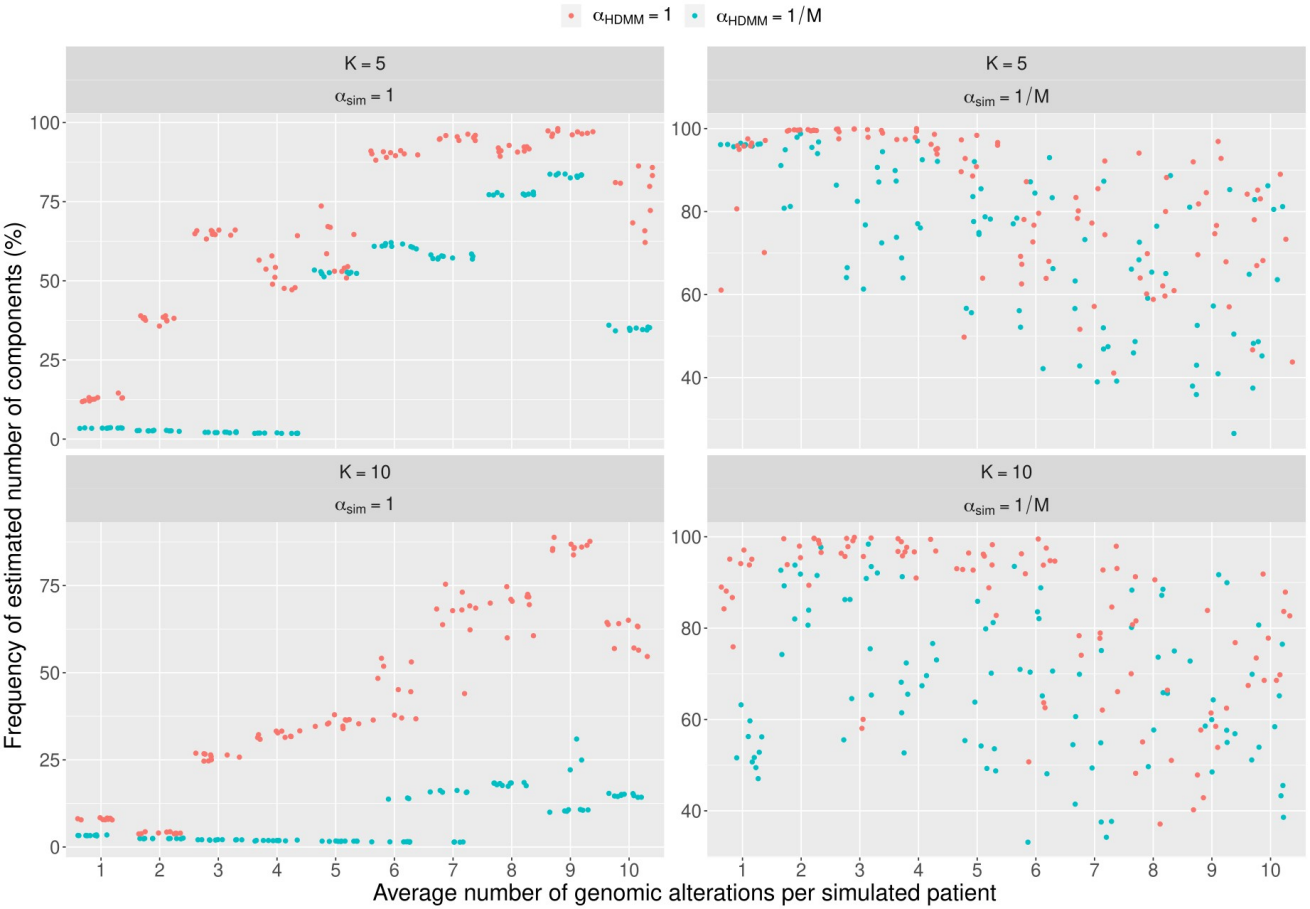
Convergence of HDMM around the number of estimated components can be hard to reach. Results on the capability of convergence of the HDMM on simulated data similar to the onco-hematological data commonly used to discover novel disease classes. In this plot we illustrate how frequently the number of components estimated by the HDMM emerges along the Markov Chain Monte Carlo (MCMC) employed to performed the fit. Exact convergence would imply 100% frequency on the y-axis. All quadrant report the number of simulated components and whether the simulated components tend to be uniform-like (*α*_*sim*_ = 1) or low-overlapping (*α*_*sim*_ = 1/*M*, where *M* is the number of genomic alterations). Plus, the color of the points represent if the HDMM was run to detect more uniform-like components (*α*_*HDMM*_ = 1, in red) or more disjunct components (*α*_*HDMM*_ = 1/*M*, in blue). The greater is the frequency up the y-axis, the closer the HDMM is to convergence.

The convergence of the eighty HDMMs was analyzed by first looking at the difference between the expected and the observed number of components, and then at the frequency of such number along two-hundred thousand MCMC iterations. The number of estimated components was considered as the most popular number along the MCMC steps. Fig 2 shows that for *α*_*sim*_ = 1 the HDMM driven by *α*_*HDMM*_ = 1 seemingly converges to *K* uniformly along the average number of genomic alterations per simulated patient. This holds as well for *α*_*HDMM*_ = 1/*M* only when the average number is greater than four for *K* = 5 and greater than seven for *K* = 10. Under *α*_*sim*_ = 1/*M*, though, the number of estimated components seems to linearly increment along the average number of alterations for both *α*_*HDMM*_ values and is always higher when *α*_*HDMM*_ = 1/*M*. These results are complemented by Fig 3 that reports how frequent the estimated number of components are along the fit. When *α*_*sim*_ = 1 the frequency raises quickly for increasing number of average alterations in the case of *α*_*HDMM*_ = 1 but, only after a certain threshold in the other case *α*_*HDMM*_ = 1/*M*. Besides, with *K* = 10 the frequency slowly increases for *α*_*HDMM*_ = 1/*M*. A different scenario is portrayed by the test on *α*_*sim*_ = 1/*M*, where frequency slowly drops for larger average number of genomic alterations per simulated patient but it is still higher than many cases w.r.t. *α*_*sim*_ = 1. Through all tests results modelled by *α*_*HDMM*_ = 1 show to achieve higher frequencies w.r.t. *α*_*HDMM*_ = 1/*M*, although only marginally if *α*_*sim*_ = 1/*M*.

### Novel stratification of simulated patients

**Fig 4:**
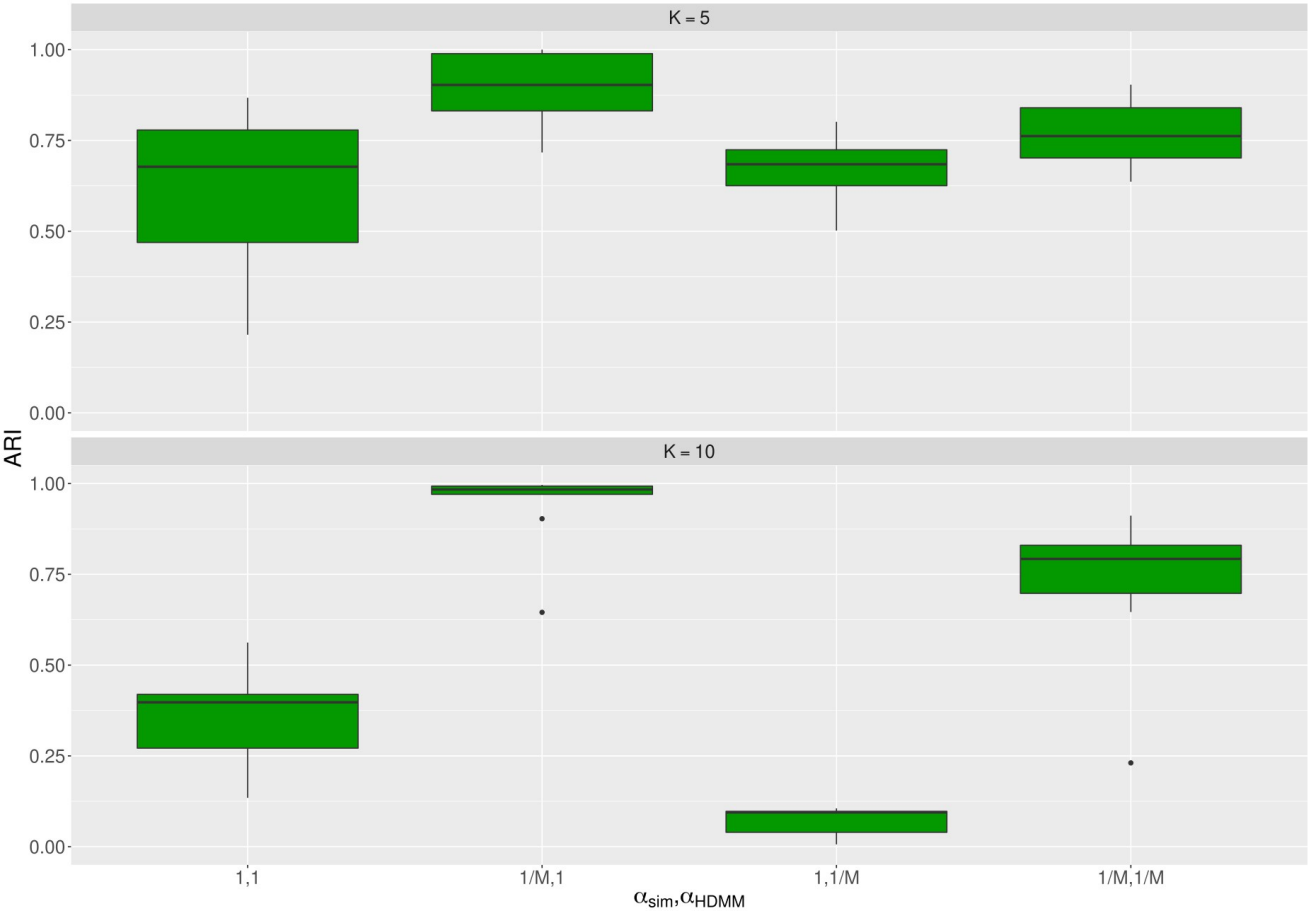
Stratification performance of multinomial-based approach on simulated patients. Illustration of the accuracy of our proposed maximum-likelihood approach based on multinomials to assign the simulated patients to the HDMM components. The metrics of accuracy is the Adjusted Rand Index (ARI), which is able to deal with scenarios where the observed number of components was found different from the expected one. ARI equals to one matches perfect agreement. The upper quadrant reports the result for *K* = 5 simulated components, while the lower quadrant does it for *K* = 10 components. The variables *α*_*sim*_ and *α*_*HDMM*_ respectively indicate when the expected components were uniform-like simulated (*α*_*sim*_ = 1) or were low-overlapping (*α*_*sim*_ = 1/*M*). Similarly, scenarios with *α*_*HDMM*_ = 1 indicate when the HDMM was set to find poorly disjunct components, whereas *α*_*HDMM*_ = 1/*M* caused the HDMM to estimate highly disjunct components. The boxplots in the plot summarizes the performance across any average number of genomic alterations per simulated patient.

As introduced above, the outcome of a HDMM is a matrix counting the number of times a genomic alteration is clustered to the components. We used the clustering result to characterize each component as a multinomial distribution by estimating their parameters. Then we utilized the probability mass function (p.m.f.) of the multinomials to stratify the simulated patients. Since we knew which component had yielded each simulated patient, we performed stratification with our maximum-likelihood based approach and then we quantitatively measured the accuracy of the stratification. Using the Adjusted Random Index (ARI) [21] as metrics for accuracy, the herein proposed approach achieves median performances roughly above 0.60 for all four combinations of *α*_*sim*_ and *α*_*HDMM*_ over all averages number of genomic alterations per simulated patient for *K* = 5 (Fig 4). This is not true for *K* = 10 where performances are maintained high only for *α*_*sim*_ = 1/*M* (median ARI above 0.79) but drop around 0.3 for *α*_*sim*_ = 1. Besides, also for the *K* = 5 the best ARI values resulted when *α*_*sim*_ = 1/*M*.

**Fig 5:**
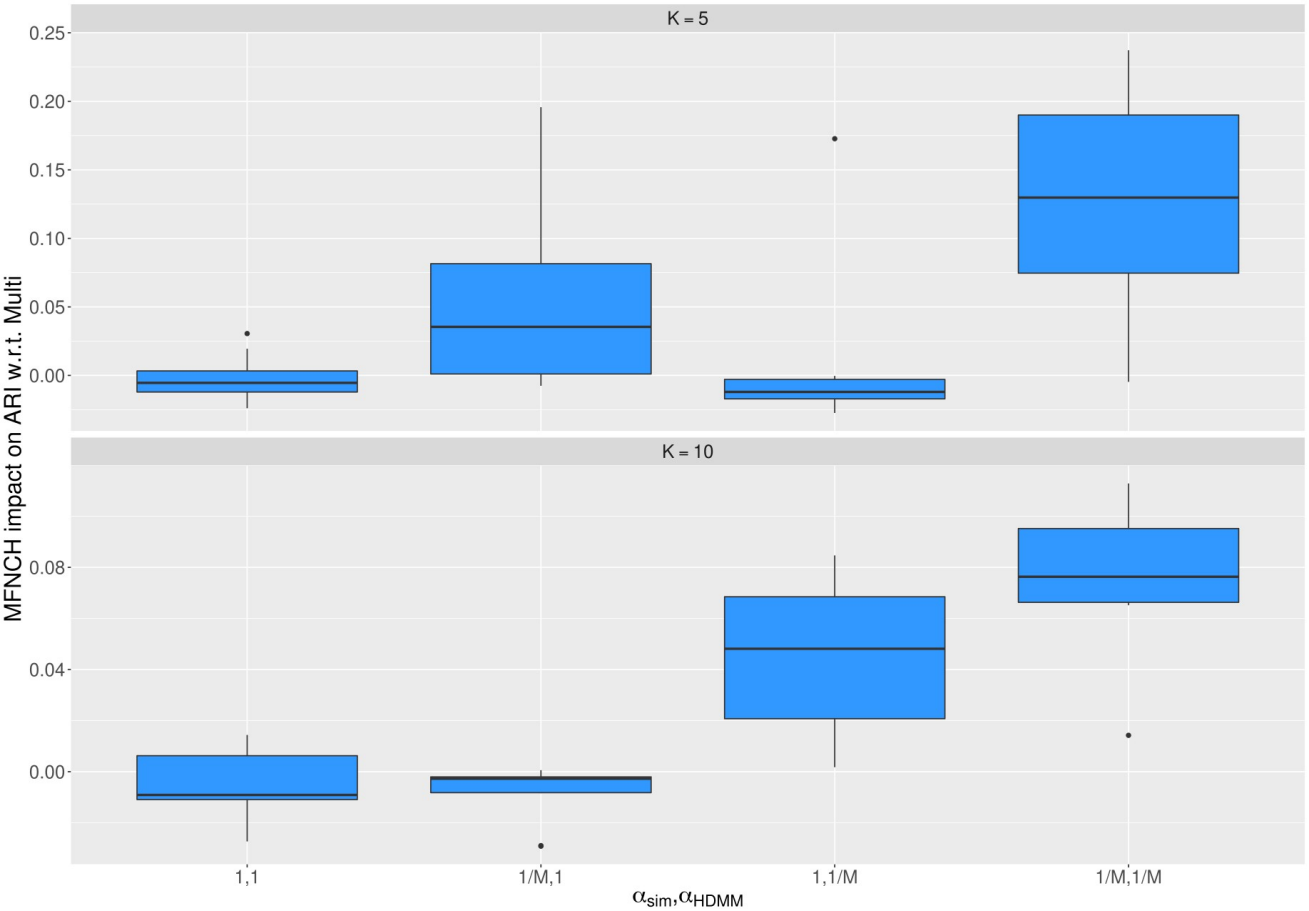
The MNFCH-based approach performs patients stratification at least as accurate as the multinomial-based approach. Overview of the impact on accuracy of using the MFNCH-based approach instead of the multinomial-based approach to characterize the components estimated by the HDMM and to assign simulated patients to such components. Each simulated patient is assigned to the component that has the highest likelihood of generating that patient. The likelihood of a component for a sample is calculated using the p.m.f. of its MFNCH distribution. The change in performance is reported for either *K* = 5 simulated components (upper) or *K* = 10 simulated components (*K* = 10). In addition, it is reported for all combination of the concentration parameters *α*_*sim*_ and *α*_*HDMM*_, which respectively regulate whether the simulated components tend to be conjuct (*α*_*sim*_ = 1) or disjunct (*α*_*sim*_ = 1/*M*) and if the HDMM was run to fit a mixture of more uniform-like (*α*_*HDMM*_ = 1) or low-overlapping components (*α*_*HDMM*_ = 1/*M*). A positive value on the y-axis reflects an uplift of accuracy when stratifying simulated patients.

We also propose to characterize the components as MFNCH distributions from the HDMM outcome. This alternative way also brings a new option to perform patients stratification across the components. In our analysis, the parameters for the MFNCH components, along with their partition functions, were estimated and the simulated patients were assigned to their most likely component. In other words, a patient gets assigned to the component that has the largest likelihood of generating that patient. As for our previous multinomial-based approach the likelihood being calculated from the p.m.f. of the MFNCH distribution. Fig 5 shows the relative difference of accuracy for the MFNCH-based apporach w.r.t. to the multinomial-based approach. The figure illustrates that when the performance of the MFNCH-based approach does not improve, it is never greatly lower than the alternative multinomial-based approach. In fact, the median measured negative ARI gap of this clustering is lower than 0.02. Differently, when the MFNCH-based approach has a positive impact, it uplifts the performance more strongly. Namely, for *K* = 5 and *K* = 10 the median gain in performance when HDMM with *α*_*HDMM*_ = 1/*M* is employed to fit simulated data being generated using *α*_*sim*_ = 1/*M* was on average 0.1 and at most was greater than 0.2. Other additional slight uplifts can be noticed for *K* = 5, *α*_*sim*_ = 1/*M* and *α*_*HDMM*_ = 1; and for *K* = 10, *α*_*sim*_ = 1 and *α*_*HDMM*_ = 1/*M*.

### Comparison of genomic prioritizations in AML

To showcase the potential impact of using either the multinomial or MFNCH based approaches for clustering, the outcome of a fitted HDMM on real onco-hematological data was taken into account (see S1 Table). Therefore we did not re-run the HDMM but we exclusively dedicated to the second part of the standard workflow. In this study, the HDMM was used to model the genomic data of 1540 AML patients and was eventually utilized to propose a new clinical disease classification. For every patient the presence or absence of 84 genomic alterations, which included gene mutations and cytogenetics anomalies, was provided. The standard workflow generated ten components from the HDMM, that later became eleven after proper adjustments due to a priori clinical knowledge. The final components were proposed as AML types characterized by driving genomic alterations that either were found frequent in the component or carried clinical relevance. Then, patients were stratified across components depending on the genomic drivers they carry. Patients carrying multiple drivers belonging to different AML components were considered ambiguous cases and ultimately not assigned.

We characterized the final components both as multinomials and MFNCH distributions. The multinomial scenario is the most statistical rigorous approach since the HDMM was essentially run to fit a multinomials mixture, while the MFNCH-approach introduces a new perspective on the genomics panorama of the disease. Table 1 compares the three diverse prioritizations. Along with the rank of the top six non-zeros genomic alterations to prioritize, the table reports the number of times an alteration was clustered into a component. The multinomial-based approach indicates, in the left column, that the most frequently clustered genomic alterations are also the most prioritized, as being in the top rank. Differently, the standard workflow of the previous study, reported in the central column, identified only a few genomic alterations for each component that are considered to be genomic drivers. In particular, component 6 was divided by study in two components with well-distinct drivers, i.e., t(15;17) and t(6;9), based exclusively on clinical considerations. Here, component 6 was held together. Additionally, except for components 2 and 3, the other eight components were characterized by a single genomic driver. The drivers in the central column render genomic alterations that are frequently clustered to the components but that do not correspond always to the most frequent genomic alterations. This is clear for components 6, 8, and 9 from Table 1, where, respectively, t(15;17) and t(6;9), t(x;11q23) and inv(3) were not the most frequent alterations in their component. The reason to choose them was motivated by a priori clinical knowledge, which plays a pivotal role in the standard workflow. The right column exhibits the prioritization provided by the usage of the MFNCH-based approach. With this characterization, the first top genomic alterations coincides with a genomic driver of the standard workflow in all components, differently from the multinomials prioritization. It is clear then that the multinomial-based and MFNCH-based approaches can prioritize alterations very differently. Among all, component 2 exhibits the most neat discrepancy since, with the MFNCH-based approach, mutations previously reported as being very frequent, such as *ASXL1* and *RUNX1*, become low-ranking and almost the entire multinomial prioritization changes. On the other hand, prioritizations can also be very similar, though, as pointed out by component 7, which is the only component whose top three prioritized genomic alterations are exactly the same in both characterizations. In general, in most components, i.e., components 1, 3, 4, 5 and 10, the first top genomic alteration does not change between the multinomial and MFNCH characterizations and it also matches the genomic driver (or drivers) defined by the standard workflow. These findings on different prioritizations inevitably suggest that patients stratification can be diverse between the three approaches, with no well-defined objective way to ascertain which performs best. It is however noteworthy to underline that the MFNCH-based approach prioritized all the genomic drivers proposed by the standard workflow in all components, as this is an achievement that the multinomial-based approach failed.

**Table 1:** Characterizations of the HDMM components on Acute Myeloid Leukemia (AML) from the three different approaches. Comparison of the three approaches used on a public AML dataset in terms of how components are characterized after the HDMM fit. The left column indicates our multinomial-based approach that is the most rigorous statistical approach, given that the HDMM is run to estimate a mixture of multinomials. In this case, each component is considered as a multinomial and the most frequent genomic alterations are prioritized. The central column reports the driving genomic alterations chosen by the usual standard workflow in onco-hematology. The driver alterations are chosen based on how frequent they are associated with the component and on a priori clinical knowledge. The right column exhibits the prioritization provided when characterizing each component as a MFNCH distribution. This latter seems to show the best compromise between a pure statistical approach (i.e., multinomial-based) and an clinically educated one (i.e., standard workflow). Bold genomic alterations indicate the driving genomic alterations reported by the standard workflow (central column). In both left and right columns only the top six alterations with non-zeros parameters are reported. Plus, beside each alteration the number of times that alteration was clustered by the HDMM into a component is reported. The vertical bar for components 2 and 3 in the central column is a logic OR between drivers, i.e., they are equally prioritized.

	Multinomial prioritization	Standard workflow prioritization	MFNCH prioritization
1	***NPM1*** (436), *DNMT3A* (304), *FLT3^ITD^* (239), *TET2* (99), *NRAS* (93), *PTPN11* (86), etc.	***NPM1***(436)	***NPM1*** (436), *MYC* (27), *DNMT3A* (304), *RAD21* (44), *IDH1* (78), *PTPN11* (86), etc.
2	***RUNX1*** (109), ***SFRS2*** (89), ***STAG2*** (65), ***MLL*** *^PTD^* (64), ***ASXL1*** (56), *TET2* (53), etc.	***RUNX1*** (109) | ***SFRS2*** (89) | ***STAG2*** (65) | ***MLL*** *^PTD^* (64) | ***ASXL1*** (56)	***STAG2*** (65), *ZRSR2* (12), ***SFRS2*** (89), ***MLL*** *^PTD^* (64), ***RUNX1*** (109), ***ASXL1*** (56), etc.
3	**Complex karyotype** (161), **-5/5q** (107), ***TP53*** (98), **-17/17p** (77), -7 (50), abn(3q) (43), etc.	**Complex karyotype** (161) | **-5/5q** (107) | ***TP53*** (98) | **-17/17p** (77) | -7 (50) | **-12/12p** (42) | **abn(7)** (17)	**Complex karyotype** (161), abn(3q) (43), **-17/17p** (77), **abn(7)** (17), **-5/5q** (107), ***TP53*** (98), etc.
4	**inv(16)** (82), *NRAS* (66), *FLT3^TKD^* (23), +8/8q (21), +22 (18), *KIT* (18), etc.	**inv(16)** (82)	**inv(16)** (82), +22 (18), *KIT* (18), *NRAS* (66), -7q (10), +21 (7), etc.
5	***CEBPA*** *^biallelic^* (72), *GATA2* (33), *NRAS* (32), *WT1* (28), *CEBPA^monoallelic^* (14), *EP300* (13), etc.	***CEBPA*** *^biallelic^* (72)	***CEBPA*** *^biallelic^* (72), *GATA2* (33), *EP300* (13), *WT1* (28), *CEBPA^monoallelic^* (14), -9q (11), etc.
6	*FLT3^ITD^* (70), **t(15;17)** (65), *WT1* (44), +8/8q (29), *FLT3^TKD^* (21), t(6;9) (14), etc.	1. **t(15;17)** (65) 2. **t(6;9)** (14)	**t(6;9)** (14), **t(15;17)** (65), *WT1* (44), *FLT3^ITD^* (70), +8/8q (29), *FLT3^other^* (14), etc.
7	**t(8;21)** (63), -Y (39), *KIT* (36), -9q (17), *RAD21* (8), *EZH2* (7), etc.	**t(8;21)** (63)	**t(8;21)** (63), -Y (39), *KIT* (36), -9q (17), JAK2 (2), *EZH2* (7), etc.
8	+8/8q (16), **t(x;11q23)** (14), -4/4q (1)	**t(x;11q23)** (14)	**t(x;11q23)** (14), +8/8q (16), -4/4q (1)
9	-7 (38), *NRAS* (27), **inv(3)** (23), *PTPN11* (21), *KRAS* (21), *RUNX1* (15), etc.	**inv(3)** (23)	**inv(3)** (23), *ETV6* (12), *SF3B1* (13), -7 (38), *KRAS* (21), *PHF6* (12), etc.
10	***IDH2*** *^R172^* (36), *DNMT3A* (27), +8/8q (10), *CREBBP* (10), -7q (8), BCOR (6), etc.	***IDH2*** *^R172^* (36)	***IDH2*** *^R172^* (36), *CREBBP* (10), -7q (8), *BCOR* (6), *DNMT3A* (27), *MLL*^PTD^ (5), etc.

## Discussion

We built this work upon a well-accepted workflow in onco-hematology that leverages on HDMMs in order to better stratify patients based on genomic alterations (i.e., presence or absence of both gene mutations and cytogenetics anomalies). Most of the commonly observed genomic alterations already contribute to outline disease subtypes according to the World Health Organization [22], which support clinicians to improve diagnostic and prognostic precision. Therefore, our focus lies on a well-defined clinical problem in onco-hematology. The standard workflow is mainly organized in two parts. Firstly, a HDMM of multinomials is employed to cluster the genomic alterations of all patients into multiple components, whose number is also unsupervisedly estimated by the HDMM. Secondly, the HDMM components are characterized in order to use them to stratify patients. Usually, a single genomic driver, or a small subgroup, is chosen to characterize each component. The choice of the drivers is motivated by how they cluster into the components and what clinical relevance they carry. Here, we show alternative statistical approaches to carry out the second part of the workflow that can enhance its applicability. Additionally, given that the first part of the workflow crucially clusters genomic alterations and automatically detects the number of components, we first show on simulated data that the convergence of the HDMM is not always guaranteed and must be taken care of to avoid inaccurate results. Our simulated patients attempted to reproduce the usual genomic panorama of onco-hematological patients in terms of number of average alterations per patient and of the underlying number of components they are supposed to derive from. The convergence of the HDMMs was evaluated on ten thousand samplings along two-hundred thousand MCMC iterations. Along iterations, the number of estimated components changes as well as their estimated parameters but, upon ideal convergence, both this number and the parameters should stabilize. In other words, we expect the variance of the number of estimated components to decrease as the MCMC iterations move forward and we expect that number to match the expected *K* number of simulated components. Therefore, the most frequent number of components over MCMC iterations was considered as the best estimate for *K* and its frequency quantified its statistical fluctuation. The results showed different convergence behaviors along the average number of alterations per simulated patient between expected distributions simulated with *α*_*sim*_ = 1 and with *α*_*sim*_ = 1/*M*. In the first case, when simulated components have similar and uniform parameters, higher 289 average number improves convergence, especially for *α*_*HDMM*_ = 1. Interestingly, if *α*_*HDMM*_ = 1/*M*, the number of components increases and its frequency is low until a certain average number of simulated alterations per patient, between five and seven, where both metrics abruptly improve. These results for *α*_*HDMM*_ = 1/*M* highlight that many similar expected components are better recognized after a certain average number of total observations. Differently, when *α*_*sim*_ = 1/*M*, both *α*_*HDMM*_ values indicate that lower average of total alterations results in a better convergence. Intuitively, this scenario suggests that when the expected components are more disjunct, i.e., they are extremely concentrated on a few well discriminant alterations, they can be recognized when only when the average number of alterations per simulated patient is low. In fact, when the average number of alterations is high, simulated patients from a component do not only carry their very discriminant alterations but also rare alterations, which add noise to the HDMM fitting process. The results on convergence also express two main concepts: the scenario with *α*_*HDMM*_ = 1 is more likely to converge but it is more conservative, i.e., little number of components; the scenario with *α*_*HDMM*_ = 1/*M* does not always converge (even lower than 50%), but when it does, it may in-depth describe the expected components (with a risk of overestimate their number). These results in the context of onco-hematology can help (i) to control the quality of the HDMM outcome, as preventing to identify an excessive number of estimated components, (ii) to adjust pre-processing (e.g., removing very rare genomic alterations) to facilitate the fit of the HDMM and (iii) to deduce whether the supposed underlying components appear to be more uniform-like or more disjunct. We remark that post-processing on the HDMM outcome might be implemented to manage its quality in terms of addressing possible noisy or highly variable components. Besides, our comments were based on the results emerging after a fixed reasonable number of MCMC iterations, which can be easily increased at the price of a more time-consuming run. We provide these observations on convergence to improve the efficacy of the HDMM to robustly cluster genomic alterations prior to using its outcome for components characterization and eventually patients stratification. Moreover, we assess on the same simulated data how our two proposed approaches to carry out the second part of the workflow perform. That is, we characterize each component either as a multinomial or a MFNCH distribution, and then we assigned each simulated patient to the component that most likely can yield it. By doing so, patients are assigned to a component based on their presence or absence of all genomic alterations, and not only on a few driver alterations, which is what the standard workflow does. Since the standard workflow cannot not be run on simulated data because they have no clinical value, we calculated the ARI between the expected and estimated components for both the multinomial-based and the MFNCH-based approach. This metric was chosen to quantify accuracy because the number of expected and observed components may differ. We firstly observed the performance of the multinomial-based approach since it represents what the HDMM fit worked with. In general, the best performances are achieved when patients were simulated by components with *α*_*sim*_ = 1/*M*. This result aligns with what previously observed in the convergence study, because the components are almost disjunct and differ the most. In contrast, when they are similar uniform (*α*_*sim*_ = 1) ARI metrics drop and seem to decrease particularly when *K* is larger. This can be explained by the lack of strong discriminants between the simulated components. In summary, strong different patterns of genomic alterations can be captured by the HDMM and their patients are accurately assigned by an intuitive statistical approach based on the usage of the multinomial likelihood. To our knowledge this multinomial-based approach for patient stratification was never directly utilized in the workflow used in onco-hematology. We secondly used the alternative MFNCH-based approach to perform stratification of the simulated patients and we could appreciate its mostly positive benefits w.r.t. the usage of multinomials. Namely, the results interestingly showed that the ARI metrics for the MFNCH-based approach are approximately at least as accurate as the ones obtained by the multinomial-based approach. Besides, a clear boost is achieved in the scenario with *α*_*sim*_ = *α*_*HDMM*_ = 1/*M*. This scenario emerges when the simulated patients derived from more disjunct components (*α*_*sim*_ = 1/*M*) and are modelled by a HDMM that was set to find more disjunct components (*α*_*HDMM*_ = 1/*M*). Beneficial effect of the MFNCH-based approach can also be observed in other scenarios, but it is essential to notice that there is no simulated situations where the multinomial-based approach significantly outperforms the it. This result suggests that the MFNCH-based is able to characterize components as good as multinomials, if not better. This can be explained because the MFNCH distributions can highlight poorly frequent genomic alterations that are biasedly clustered into specific components, which is particularly crucial for rare alterations. This enriched clustering remains unseen by multinomial-based approach because it is exclusively based on frequency.

Our analysis on real data supported these observations on simulated data and extended the comparison including also the result from the standard workflow in onco-hematology. The data in this case were provided by a study on more than one thousand AML patients. Since the outcome of the HDMM was publicly available we could focus exclusively on the second part of the workflow, where we applied our proposed approaches. We appreciated that the final characterizations of the components were mostly similar but with slight and outstanding differences. The multinomial-based approach showed to be extremely rigorous in prioritizing alterations in components, ranking them from the most frequently clustered to the least one. The standard clustering implicitly leveraged on the multinomial prioritization but adjusted it based on a priori clinical knowledge when choosing the genomic driver to prioritize. This is especially true for three components, where the chosen drivers were not the top frequent alterations in the components but were picked regardless due to their clinical importance. These were the cases for t(15;17) and t(6;9), inv(3) and t(x;11q23). The outstanding result was that the MFNCH-based approach characterized the components such that even the driving genomic alterations for these three components were firstly prioritized. Therefore, this approach spontaneously highlights those alterations known to carry clinical relevance. This good trade-off might suggest that other alterations prioritized by the MFNCH-based approach might harbor a clinical value unseen so far. Except for the three components where the multinomial-based approached failed to clearly highlight the driving alterations identified by the standard clustering, the MFNCH-based prioritization on most the other components aligns with both the usage of multinomials and with the drivers of the standard workflow. This hints at the MFNCH-based approach being at least as good as if not better at characterizing components than the other two approaches. Of course, the usage of the MFNCH implies some critical adjustments. For example, very rare genomic alterations in the cohort may turn out as being extremely relevant according to the MFNCH-based approach if not properly handled. In addition, the utility of the MFNCH-based approach to assign patients to components depends on the difficulty of calculating the partition functions of such components. Differently from the multinomials, the partition functions of the MFNCH distribution depends on its parameters and its calculation can be extremely time demanding. Despite these possible shortcomings, the results of the MFNCH-based approach on real AML data showed to find a good trade-off between the rigorous usage of the multinomials and the common standard workflow that leverages on a priori clinical knowledge.

## Conclusion

The herein presented approaches ultimately intend to contribute to enhance patients stratification for use in clinical practice. We showed on both simulated and real data that the MFNCH-based approach can efficiently contribute to the current standard workflow in onco-hematology and can provide a novel way to characterize the genomic panorama of onco-hematological diseases. New classification systems in onco-hematology may benefit from the insights given by the MFNCH-based approach, which can help clinicians to further tailor patients stratification and ultimately personalized treatments.

## Supporting information

S1 Data Supporting materials and methods. This file contains sections deep diving in the detailed aspects of our work from a methodological standpoint.
(PDF)

S1 Table Clustering of genomic alterations provided by the HDMM on public AML data. The table exhibits how one HDMM clusters all gene mutations and cytogenetic anomalies across one garbage component (column 0) and ten components (1-10). Some alterations are uniquely assigned to a single component but more than half are assigned at least to two components. Besides, this table shows that the genomic alterations are not equally abundant in the cohort with *NPM1* being the most frequent occurring alteration.
(CSV)
