# Supplementary material for: Covering Hierarchical Dirichlet Mixture Models on binary data to enhance genomic stratifications in onco-hematology
Source: PLoS Comput Biol. 2024 Feb 2;20(2):e1011299. doi: 10.1371/journal.pcbi.1011299 (PMC10880984; doi:10.1371/journal.pcbi.1011299)
Supplement: S1 Data — This file contains sections deep diving in the detailed aspects of our work from a methodological standpoint. (PDF) [file pcbi.1011299.s001.pdf]

Supplementary – Covering Hierarchical Dirichlet Mixture Models on binary data to enhance genomic stratifications in Onco-Hematology

S1 Appendix. Multivariate Fisher Non-Central Hypergeometric
As illustrated in Figure 1 in the main text, the multinomial distribution models the drawing process from an un urn with several marbles of different color. Each color indicates a different genomic alterations and multiple marbles with the same color can be available, which makes the most available colors the most probable to be drawn. Besides, after each draw the marble is placed back into the urn. The presence of multiple marbles of the same colors and the replacement after the draws do not match the binary nature of the onco-hematological data where the HDMM is usually employed. That is why our attention was driven towards the Multivariate Fisher Non-Central Hypergeometric (MFNCH). This distribution is one of the two multivariate non-central hypergeometric distribution: one is known as Fisher's, the other as Wallenius'. They both extend the case of the well-known multivariate hypergeometric distribution, which models the sampling-without-replacement scenario. Given M features and $C_1, C_2, ..., C_M$, where C_m indicates the number of maximal draws for the m-th feature, the hypergeometric distribution follow:

$$P(x_1, x_2, ..., x_M) = \frac{\prod_{m=1}^{M} \binom{C_m}{x_m}}{\binom{N}{n}} \ , \tag{1}$$

where $\sum_{m=1}^{M} C_m = N$ and n is the number of total draws. Fisher's and Wallenius' distributions are different from a theoretical standpoint and their application change based on the assumption of the targeted observations. If one wants to model the draws one at a time, then the Wallenius' non-central distribution should be used. Otherwise, when the draws are assumed to be independent, as we did, the process can be modelled by the Fisher's non-central distribution. The probability mass function (p.m.f.) of the Fisher's non-central distribution becomes then:

$$P(x_1, x_2, ..., x_M; w_1, w_2, ..., w_M) = \frac{\prod_{m=1}^{M} \binom{C_m}{x_m} w_m^{x_m}}{P_0} \ . \tag{2}$$

The presence of weights w_m transforms the landscape of the draws, since a genomic alteration can be rare but with a large weight. Notably the partition function of the MFNCH distribution can be hard to compute. Yet, it is crucial when comparing two distributions with different weights. This is not the case for multinomials, whose p.m.f.

$$P(x_1, x_2, ..., x_M; p_1, p_2, ..., p_M) = \frac{\Gamma(\sum_{m=1}^{M} x_m + 1)}{\prod_{m=1}^{M} \Gamma(x_m + 1)} \prod_{m=1}^{M} p_m^{x_m} \ . \tag{3}$$

easily shows that the partition function does not depend on its parameters. In conclusion, the MFNCH distribution can model extractions from an urn containing many marbles but each with a different color and with no replacement after the draws. This matches the binary nature of the data we are interested in since no alterations can be extracted multiple times. Besides, each marble has a different color but also a

different size, which entails that the larger is a marble, the more biased is the draw to pick it. In this way, there is one marble for each color but the bigger marbles are likely to be prioritized by the draws. We consider this distribution to be ideal to model onco-hematological data where patients are represented by binary vectors and we assume that some of their alterations should be prioritized.

S2 Appendix. Details on components extraction

In the context of onco-hematology, our work offers an approach to render the result from the Hierarchical Dirichlet Mixture Model (HDMM), commonly used to cluster genomic alterations, into an automatic tool for patients stratification. To this end, we adopted intuitive statistical considerations based on what the HDMM is utilized for and produces. The HDMM is run to fit a mixture of multinomials. As indicated in the main text of the article, the HDMM outputs a MxK matrix, with M being the number of genomic alterations and K being the number of estimated components. At the m-th row and k-th column, the matrix contains the number of times the m-th genomic alteration was assigned to the k-th component. Now, at the current status of molecular analyses in onco-hematology, this matrix is used as an insightful input to clinicians to determine a better molecular classification of the disease. Usually, if-else rules based on either the presence or absence of genomic drivers are eventually determined. Differently, our final aim is to support clinicians in decision making expressing the full statistical potential of the HDMM, which can better assist when many co-occurrent genomic alterations emerge for a patient. Since the HDMM was fitted to estimate a mixture of multinomials distributions, then the first attempt we propose is to estimate components as multinomials. Remarkably, though, the HDMM clusters genomic alterations, not patients. That is, the HDMM can and does assign genomic alterations from the same patients to different components. We are now motivated to use such components to stratify patients, as the clinically-oriented classifications do. Our second attempt we highlight is to characterize components as Multivariate Fisher Non-Central Hypergeometric (MFNCH) distributions. Meaning that, we used the counts from the final matrix of the HDMM to approximate distributions which well tailor two properties of the molecular data in onco-hematology: binarity and un-equal abundances. Although multinomials might fit binary data, they do not reproduce the binary nature of the data because they are designed to model a sampling-with-replacement scenario. Regardless, multinomials provide their parameters to prioritize genomic alterations, which definitively improve the interpretation of the components. We found the MFNCH distribution to be a suitable candidate to takeover the multinomial distribution when it comes to characterize components. This distribution models biased sampling-with-no-replacement. Given that there is no replacement, a genomic alteration can be set to be “drawn” only once (i.e., in compliance with binarity) and can be ranked according to its parameter, that express how strongly an experiment is biased to draw it. Interestingly, the presence of bias has the additional benefit of dealing with different abundances of genomic alterations in the all cohort. Namely, mutations and cytogenetic aberrations occurrences do not distribute uniformly, and those emerging frequent in the whole cohort turn out to be frequent inside components. For instance, in the real dataset we used in our test, a component includes the mutation of FLT3 and the presence of t(15;17). The mutation of FLT3 occurred in 332 patients while the translocation occurred in 65 patients in the whole cohort. Based on the result from the HDMM we can observe that only 70 FLT3 mutations were assigned to the component, but all 65 translocations were assigned to it. Under multinomial distribution we are driven to prioritize that FLT3ITD over t(15;17) since it is more frequent, but clearly there was no chance to have more than 65 counts for t(15;17). Intuitively, we are drawn to think that t(15;17) is enriched in this components, while FLT3 simply maintain its dominant

presence given how frequent it is. This is where the MFNCH can intervene and help changing priorities inside components. Further details can be found in the next sections.

S3 Appendix. Details on HDMM final matrix

Here, we do not report the fitting procedure of the HDMM since it has been extensively explained in literature and is not part of the scope of this work. Though, to understand how the HDMM outcome looks like we mentioned in the main text that a Markov chain Monte Carlo (MCMC) was used and that a matrix is generated every step of the MCMC chain. In this section we want to break down our post-processing workflow that was fed by the outcome of the HDMM fit (i.e., the outcome of the MCMC). In all our tests, for a single chain, we "burn" the first 5000 samplings, then we run the chain to get other two-hundred thousand samplings and we preserve one matrix every 20 samplings. Therefore, we end up with ten thousand matrices. In other words, we have ten thousand instances of how genomic alterations distribute across components. Since the number of components can vary, especially when convergence is hard to accomplish, the number of the columns in the matrices changes. Plus, the order of the components in the matrix is not necessarily guaranteed. As reported in the main text, we consider the most frequent number of components over such ten thousand iterations as the best estimate for the true number of components. Thus, to estimate the components we decide to use only those iterations with a number of column equal to the best estimate. Doing so, the chosen matrices share their size. Next, we normalize the matrices column-wise so that each column (i.e., component) has values whose sum is one. To get one final single matrix from a single chain we average the values across all matrices, assuming the order of columns to be same across them. Additionally, to address highly variable values across matrices, we zero out those values whose high posterior density interval overlaps 0. Therefore, the information provided by a single chain is a (genomic alterations $\times$ components) matrix derived from many iterations. Before moving on with the post-processing we filter out components with all zero values. Yet, to address possible ill starting points of the MCMC chain, we run ten independent chains for all our tests. This means that we must combine ten matrices that typically have a different number of components and whose order of components in the matrix is not shared. Even when chains show good convergence in terms of number of components, this number may change across them possibly due to either underfit or overfit. To deal with this, we assume that all chains singularly find a collection of components which can be associated to the other collections. For example, a chain can find two components with similar values that might be determined by another chain as a single component. To deal with this we decided to represent how components from a chain relates to components to other chains with a graph. Using cosine similarity, we calculate the pairwise distance between two components. Namely, given two chains, a component from one chain is connected exclusively to the closest component from the other. In this way we were able to build a graph of components. Now, we assume that a strong component must emerge in every chain and all its representations across chains must be fully connected. Given that, we employed a modified version of the Bron-Kerbosch algorithm to detect maximal cliques. All isolated maximal clique were considered final robust components because uniquely found in each chain. At the same time, we can find multiple maximal cliques to be connected (i.e., non-isolated), which typically describe the situations where a chain represented a component to be one and other chains represented it to be split in multiple ones. We count the occurrences of each representation (e.g., found one in one chain, found split in two in the other nine chains) and we test whether there is a representation of components that significantly violate the null hypothesis of all representations being equally represented. We do this with an exact goodness-of-fit test for a multinomial with equal parameters. If the

alternative hypothesis is not found to be significant, we define the connected maximal cliques as a single robust component; if not, we define one robust component for each maximal clique, despite them being connected. At this point, once the robust final components are determined, and unused components across chains are removed, we are ready to estimate each component as a statistical distribution.

S4 Appendix. Details on components parameters estimation

For the sake of simplicity, we explain our approach referring to a single component but it holds for all of them. After we realize how a single final robust component is represented in all chains, we are able to trace back all their columns in the matrices from the samplings. Additionally, we are able to collect how many patients the genomic alterations in the components where assigned from. We divide all the columns for their respective number of patients (e.g., if a mutation was assigned to the component for all patients, then its value will be one), since this number can easily change from samplings to samplings. Now, when we estimate a component as a multinomial distribution, we take the result of these divisions, we average the values across matrices and then we normalize the remaining vector of M values to one. In this way we end up with $p_{1k}, p_{2k}, \ldots, p_{Mk}$ for the k-th component. Differently, when we treat a component as a MFNCH distribution, we fit a MFNCH providing the multinomial parameters as the average "draw" (i.e., $\bar{x}_1, \bar{x}_2, \ldots, \bar{x}_M$) and the number of occurrences of each genomic alteration in the whole cohort as the abundances ($C_{m m=1,..,M}$). Doing so, we are able to determine the weights $w_1, w_2, \ldots, w_M$, which enable us to prioritize alterations in each component.

S5 Appendix. Details on patients stratification

Once components are characterized by a statistical distribution, we utilize the probability mass functions to assign every patient. For multinomial distribution, that is:

$$\kappa_i = \underset{k}{\operatorname{argmax}} \prod_{m=1}^{M} p_{mk}^{x_{im}} \tag{4}$$

which holds for the i-th patient. We remark that the partition function is the same across all components, therefore we can avoid computing it. For MFNCH distribution the p.m.f. formulation eases because we set all C_m to one. With that said, we perform stratification for the i-th patient with:

$$\kappa_i = \underset{k}{\operatorname{argmax}} \frac{1}{P_{0k}} \prod_{m=1}^{M} w_{mk}^{x_{im}} \ , \tag{5}$$

where we can manage to exactly calculate P_{0k} since the number of genomic alterations is not large.
